# A Potentiometric Flow Biosensor Based on Ammonia-Oxidizing Bacteria for the Detection of Toxicity in Water

**DOI:** 10.3390/s130606936

**Published:** 2013-05-24

**Authors:** Qianyu Zhang, Jiawang Ding, Lijuan Kou, Wei Qin

**Affiliations:** 1 Key Laboratory of Coastal Zone Environmental Processes and Ecological Remediation, Yantai Institute of Coastal Zone Research (YIC), Chinese Academy of Sciences (CAS), Shandong Provincial Key Laboratory of Coastal Zone Environmental Processes, YICCAS, Yantai 264003, China; E-Mails: qyzhang@yic.ac.cn (Q.Z.); jwding@yic.ac.cn (J.D.); ljkou@yic.ac.cn (L.K.); 2 University of Chinese Academy of Sciences, Beijing 100049, China

**Keywords:** potentiometry, flow biosensors, ion-selective electrodes, toxicity, *Nitrosomonas europaea*

## Abstract

A flow biosensor for the detection of toxicity in water using the ammonia-oxidizing bacterium (AOB) *Nitrosomonas europaea* as a bioreceptor and a polymeric membrane ammonium-selective electrode as a transducer is described. The system is based on the inhibition effects of toxicants on the activity of AOB, which can be evaluated by measuring the ammonium consumption rates with the ammonium-selective membrane electrode. The AOB cells are immobilized on polyethersulfone membranes packed in a holder, while the membrane electrode is placed downstream in the flow cell. Two specific inhibitors of the ammonia oxidation—allylthiourea and thioacetamide—have been tested. The IC_50_ values defined as the concentration of an inhibitor causing a 50% reduction in the ammonia oxidation activity have been measured as 0.17 μM and 0.46 μM for allylthiourea and thioacetamide, respectively. The proposed sensor offers advantages of simplicity, speed and high sensitivity for measuring toxicity in water.

## 1. Introduction

The detection of toxicity in water caused by pollutants is of great importance for aquatic ecosystems and human health. Bioassays are one of the most useful technologies for environmental monitoring due to their high sensitivity, good reproducibility, and easy adaptation for online measurements [1–3]. Various toxicity bioassays based on measuring the physiological responses of fish, invertebrates, plants, algae and microorganisms have been developed [4–6]. In particular, bacterial bioassays have attracted much attention because bacteria offer enormous species diversity, rapid growth rates, low cost and easy maintenance as compared to other higher organisms [7].

Ammonia-oxidizing bacteria (AOB) play an important role in the removal of ammonia for wastewater treatment [8]. They are considered to be extremely susceptible to a wide range of pollutants (*i.e.*, inhibitors) including sulfur, aromatic, and halogenated compounds even at low concentrations [9]. Therefore, AOB are well accepted target microorganisms and can be used for detecting ammonia oxidation inhibitors in wastewater. AOB are chemoautotrophic and can obtain energy for growth from the oxidation of ammonia shown as follows [10]:

(1) $${2\text{NH}}_{3}+{3\text{O}}_{2}\overset{\text{AOB}}{\to }{2\text{HNO}}_{2}+{2\text{H}}_{2}\text{O}$$

The toxicity of an inhibitor can be assessed by measuring the ammonia oxidation activity. Monitoring of oxygen consumption by microbial sensors composed of immobilized AOB cells and an oxygen electrode allows a rapid and accurate estimation of the inhibition effect on the ammonia oxidation [11,12]. However, it is necessary to distinguish the oxygen uptake by the heterotrophic substrate oxidation and endogenous respiration from that by the ammonia oxidation [7]. By measuring the nitrite formation rates with the colorimetric methods using cell suspensions, the inhibitor toxicity can also be evaluated [13–15]. However, the analysis procedures are always complex and time-consuming [16]. Furthermore, the reagents used are harmful to human health and may contaminate the environment. Therefore, new methods for convenient detection of ammonia oxidation activity of AOB are highly required.

Polymeric membrane ion-selective electrodes (ISEs) offer advantages of excellent selectivity, low cost, ease of use, and high reliability, and have been successfully used for analysis of water quality [17,18]. In this work, we employ *Nitrosomonas europaea* (*N. europaea*) as an AOB model and an ammonium-selective membrane electrode as a transducer for measuring the inhibition effect of a toxicant on the AOB ammonia oxidation activity. Unlike the conventional biosensing scheme in which a bioreceptor is usually immobilized on the surface of an electrode or optrode, the proposed system allows the molecular recognition and transduction processes to be done individually. The AOB cells are immobilized on polyether sulfone membranes packed upstream in a holder, while the membrane electrode is placed downstream in the flow cell. The flow biosensing mode simplifies the sensor construction and permits one to execute the individual unit operations under optimum conditions rather than to operate them concurrently under compromised conditions [19].

## 2. Experimental Section

### 2.1. Materials

*N. europaea* (NBRC 14298) was purchased from the NITE Biological Resource Center, Chiba, Japan. The ammonium ionophore (nonactin), potassium tetrakis(4-chlorophenyl)borate (KTClPB), 2-nitrophenyloctyl ether (*o*-NPOE), high molecular weight polyvinyl chloride) (PVC), *N*-2-hydroxyethylpiperazine-*N'*-2-ethanesulfonic acid (HEPES) and Trizma base were purchased from Sigma-Aldrich (St. Louis, MO, USA). All chemicals and reagents were of selectophore or analytical reagents grade. Aqueous solution were prepared with freshly deionzed water (18.2 MΩ cm specific resistance) obtained with a Pall Cascada laboratory water system.

### 2.2. Electrode Preparation

The ammonium-sensitive membrane contained 1.0 wt% nonactin, 0.3 wt% KTClPB, 32.9 wt% PVC and 65.8 wt% *o*-NPOE [20]. The membrane was obtained by casting a solution of ∼125 mg of membrane components dissolved in 2.0 mL tetrahydrofuran (THF) into a glass ring of 2.0 cm diameter fixed on a glass plate and letting the solvent evaporate overnight. For each electrode, a disk of 6 mm diameter was punched from the membrane and glued to a plasticized PVC tube (i.d. 4 mm, o.d. 6 mm) with THF. The internal filling solution was 10^-2^ M NH_4_Cl and the electrode was conditioned in 10^-2^ M NH_4_Cl for 1 day. When not used, the electrode was placed in the conditioning solution.

### 2.3. Cultivation and Immobilization of N. europaea

*N. europaea* cells were cultured in a growth medium (pH 7.8) consisting of 18.90 mM (NH_4_)_2_SO_4_, 3.67 mM KH_2_PO_4_, 5.95 mM NaHCO_3_, 400 μM MgSO_4_·7H_2_O, 30 μM CaCl_2_·2H_2_O, 180 μM Fe-EDTA, and 50 mM HEPES. The cells were shaken in the dark at 28 °C. When the bacteria were grown to the late logarithmic phase, the cells were collected by vacuum filtering with a polyethersulfone membrane (diameter: 25 mm, pore size: 0.2 μm, Pall Corporation, Ann Arbor, MI, USA). After the cells were adsorbed on the membrane, another polyether sulfone membrane was placed over the cells to make a sandwich form. Then the sandwich form was fixed with an O-ring on a 25 mm membrane holder (Pall Corporation, Ann Arbor, MI, USA). When not in use, the cells were stored at 4 °C in the growth medium.

### 2.4. Apparatus

**Figure 1.**
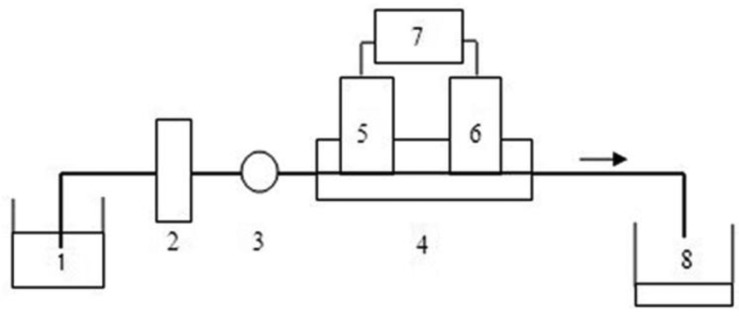
Schematic diagram of the flow monitoring system: (1) sample solution; (2) peristaltic pump; (3) membrane holder; (4) flow cell; (5) ammonium-selective electrode; (6) reference electrode; (7) ion analyzer; (8) waste.

Figure 1 shows the schematic diagram of the flow monitoring system. The propulsion of the solution was accomplished with a peristaltic pump (IFIS-D, Xi'an Remex Analyse Instrument Co., Ltd., Xi'an, Shaanxi, China). The membrane holder with immobilized cells was placed between the pump and the flow cell. The detection chamber was constructed in-house from a single block of Perspex. An ammonium-sensitive working electrode (i.d. 4 mm, o.d. 6 mm) and an Ag/AgCl reference electrode were imbedded into the cell body with a distance of 10 mm. The whole flow-through system was assembled using Teflon tubing of a 0.8 mm internal diameter. Potentiometric measurements were performed with a Model PXSJ-216 digital ion analyzer (Shanghai Instruments Factory, Shanghai, China) in the galvanic cell: Ag/AgCl/sample solution/ISE membrane/inner filling solution/AgCl/Ag.

### 2.5. Procedures

For the control test, the peristaltic pump delivered the buffer solution (Tris-HCl buffer, 0.05 M, pH 8.0) containing 10^-4^ M NH_4_Cl at a flow rate of 1.62 mL min^-1^. When a stable potential baseline was obtained, the flow was stopped for the ammonia oxidation by the AOB cells immobilized in the membrane holder. After 30 min, the pump was started and a negative potential peak induced by the decrease in the ammonium concentration was recorded when the buffer solution passed through the ammonium-selective electrode in the flow cell. For inhibitor measurements, varying amounts of inhibitors were added to the buffer solution and delivered through the system. The additions of the inhibitors decreased the ammonia oxidation activity of the AOB cells, thus causing relatively smaller peak signals as compared to that of the control test.

### 2.6. Nitrite Assays Using Cell Suspensions

Cells were collected by centrifugation and washed with the Tris-HCl buffer several times to remove nitrite. Experiments were performed in 50 mM Tris-HCl buffer in batch reactors. Buffer solutions containing *N. europaea*, 10^-4^ M NH_4_Cl and varying concentrations of inhibitors were incubated for 30 min at 30 °C. The batch reactor without an inhibitor was run for the control. The concentrations of nitrite were measured by the colorimetric method using *N*-(1-naphthyl)ethylenediamine dihydrochloride and sulfanilic acid.

## 3. Results and Discussion

### 3.1. Optimization of the Flow Biosensing System

**Figure 2.**
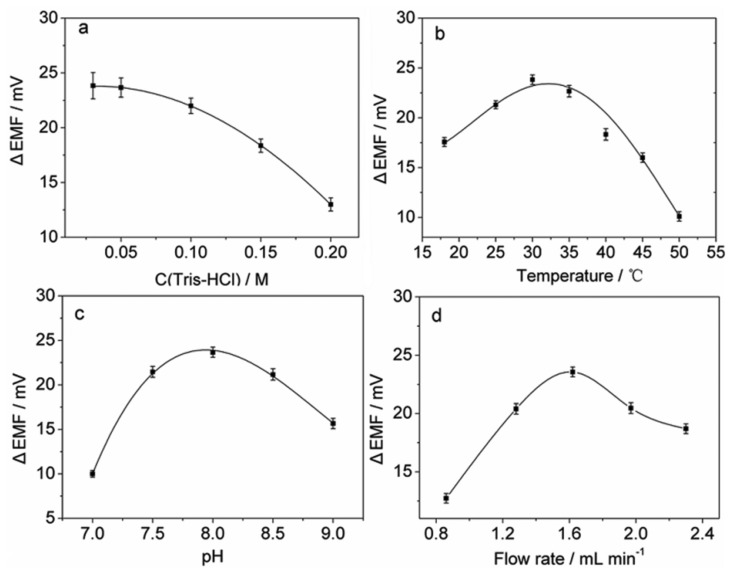
Effects of (**a**) buffer concentration, (**b**) temperature, (**c**) sample pH, and (**d**) flow rate on the potential response. Unless stated otherwise, experiments were performed under the following conditions: NH_4_Cl, 10^-4^ M; no inhibitor; buffer solution, 0.05 M Tris-HCl of pH 8.0; temperature, 30 °C; flow rate, 1.62 mL min^-1^; cell amount, 1.7 × 10^9^. Each error bar represents one standard deviation for three measurements.

The influence of the concentration of the Tris buffer was studied in the range of 0.03–0.20 M and the results are shown in Figure 2(a). It can be seen that higher buffer concentrations result in lower potential peak heights, which is probably due to the higher ion background in the buffer solution. Although decreasing the buffer concentration improves the sensitivity, it may cause larger noises in potential signals. To achieve a higher sensitivity and a lower level of noise, 0.05 M was selected as the buffer concentration.

Temperature and sample pH are key factors for most enzymatic reactions. The effect of temperature on the sensor response was investigated (Figure 2(b)). The results show that the potential response increases with increasing temperature up to 30 ° C, which is due to the increase in the enzymatic activity in the cells. Experiments also show that the response decreases with further increase in temperature probably due to the damage to the AOB cells. Therefore, 30 ° C was employed as the operational temperature for subsequent experiments. The effect of sample pH on the sensor response was examined over the range of 7.0–9.0 in 0.05 M Tris-HCl buffer. As shown in Figure 2(c), the maximum potential peak height was observed at pH 8.0, indicating that *N. europaea* cells have the highest metabolic activity at this pH value. Therefore, pH 8.0 was used for further experiments.

The influence of flow rate was studied in the range of 0.86–2.30 mL min^-1^. Figure 2(d) shows that lower flow rates may result in lower potential peak heights, which is probably due to the dispersion of the sample when flowing between the membranes with immobilized *N. europaea* cells and the flow cell with the membrane electrode. In addition, lower flow rates could prolong analytical time, and may cause peak broadening. However, on the other hand, at flow rates higher than 1.62 mL min^-1^, the sensor response decreased, which may be due to the sample dilution effect caused by vigorous mixing in the flow. Therefore, the flow rate of 1.62 mL min^-1^ was chosen for the present system.

**Figure 3.**
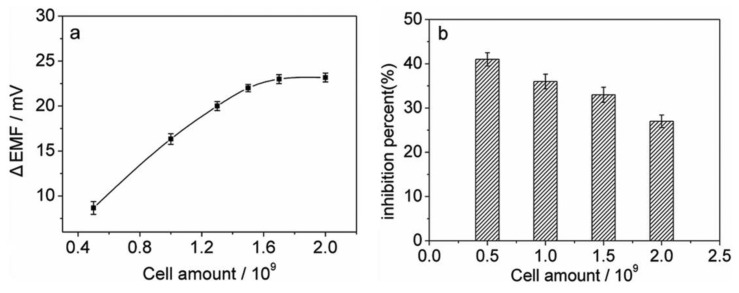
(**a**) Effect of the amount of immobilized cells on the potential response. (**b**) The inhibition sensitivities of different amounts of immobilized cells to 0.3 μM thioacetamide. The other conditions are given as in Figure 2. Each error bar represents one standard deviation for three measurements.

Experiments were performed to investigate the relationship between the sensor response and the amount of the immobilized cells. As shown in Figure 3(a), for the control test, the potential peak height increases with increasing the cell amount due to an increase in the catalytic activity of the cells, and could reach a plateau when 1.7 × 10^9^ cells are immobilized. However, the amount of immobilized cells may also affect the inhibition sensitivity of the biosensor. As shown in Figure 3(b), for measuring 0.3 μM thioacetamide, lower cell amounts could give higher sensitivities. Considering a compromise between high inhibition sensitivity and large peak height, the amount of the immobilized *N.europaea* cells was chosen as 1.0 × 10^9^ cells for subsequent experiments.

### 3.2. Inhibitory Effects of Toxicants

For the catalytic mechanism of *N. europaea*, ammonia is initially oxidized to hydroxylamine by the ammonia monooxygenase (AMO). AMO contains copper in its active site, so copper chelating agents such as allylthiourea and thioacetamide are specific inhibitors for the ammonia oxidation [21]. The presence of an inhibitor could decrease the ammonia oxidation rate (AOR). Thus, the inhibitory effect can be evaluated by comparing the inhibited AOR with that of the control. The inhibition effects of various concentrations of allylthiourea (0.05–0.5 μM) and thioacetamide (0.05–1.5 μM) were investigated. The inhibition percentages can be calculated using Equation (2):

(2) $$\text{inhibition}\;\%=\frac{\text{AOR}-{\text{AOR}}_{\text{i}}}{\text{AOR}}\times 100\%$$

where AOR and AOR_*i*_ are the ammonia consumption rates before and after exposure to an inhibitor, respectively. The concentration of ammonia can be converted from that of ammonium, while the latter can be measured by the membrane ISE according to the Nernst equation. For simplicity, the relationship between the inhibition percentage and the potential response can be described as follows:

(3) $$\text{inhibition}\;\%=\frac{10^{-F\cdot \Delta E_{i}/2.303\text{RT}}-10^{-F\cdot \Delta E/2.303\text{RT}}}{1-10^{-F\cdot \Delta E/2.303\text{RT}}}\times 100\%$$

where Δ*E* and Δ*E_i_* are the potential peak heights before and after addition of the inhibitor, and *R*, *T*, and *F* are the universal gas constant, the absolute temperature and the Faraday constant, respectively.

**Figure 4.**
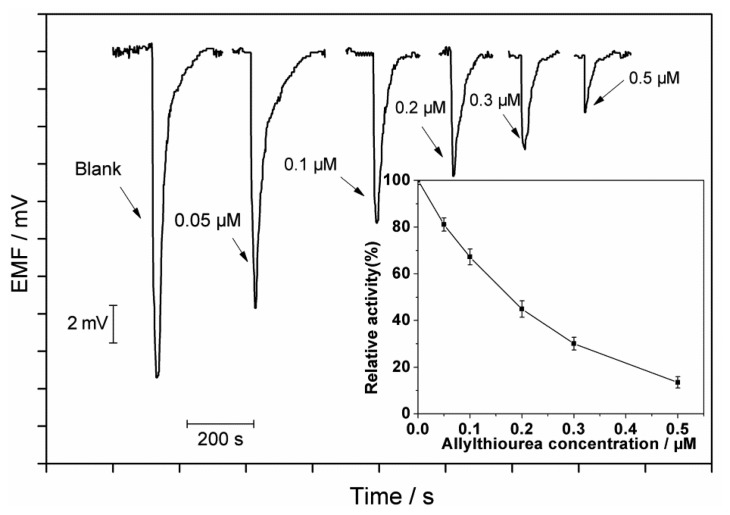
Potential responses of allylthiourea at different concentrations of 0.05, 0.1, 0.2, 0.3 and 0.5 μM. Inset shows the inhibition curve of allylthiourea (relative activity% = 100%–inhibition%). The amount of immobilized cells was 1.0 × 10^9^. The other conditions are given as in Figure 2. Each error bar represents one standard deviation for three measurements.

Figure 4 shows the inhibition effects of allylthiourea at different concentrations. It can be seen that the peak height decreases with increase in the allylthiourea concentration. The concentration of a toxic compound causing a 50% reduction in the ammonia oxidation activity from that in the control defined as IC_50_ is a useful parameter to evaluate the toxicity. The inhibition on the ammonia oxidation by an inhibitor can be expressed as follows [22]:

(4) $${\text{AOR}}_{i}=\text{AOR exp}\left({-\text{K}}_{i}C\right)$$

where K*_i_* and *C* are inhibition constant and the concentration of an inhibitor, respectively. K*_i_* and IC_50_ can be obtained by plotting ln(AOR*_i_*/AOR) *versus* inhibitor concentration C. The IC_50_ values obtained with the biosensor are 0.17 and 0.46 μM for allylthiourea and thioacetamide, respectively.

It should be noted that some substances coexisting in potable water and environmental samples might interfere with the inhibition measurements. For example, copper ions can form complexes with allylthiourea and thioacetamide and thus decrease their inhibition percentages; ammonium ions at relatively high concentrations might cause a high substrate background and make the system insensitive to the inhibitors.

### 3.3. Characteristics of the Flow Biosensor

**Figure 5.**
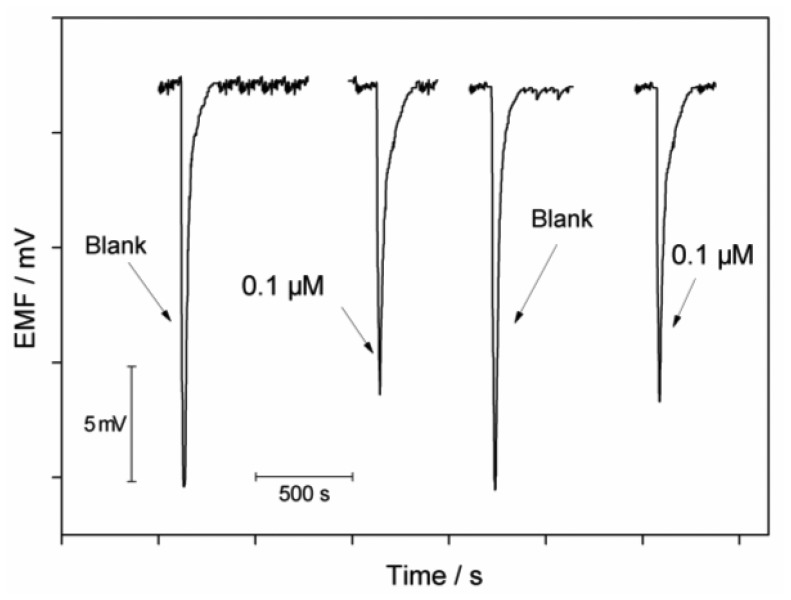
Reversibility test of the biosensor using 0.1 μM thioacetamide as the inhibitor.

The flow biosensor can be used for measurements of allylthiourea in the range of 0.05–0.5 μM and of thioacetamide in the range of 0.05–1.5 μM with the corresponding detection limits (3σ) of 0.02 μM and 0.04 μM. The precision of the system was checked with repetitive measurements of 0.1 μM of each inhibitor. The relative standard deviations are 4.0% and 4.4% for allylthiourea and thioacetamide, respectively (n = 4). Reversibility of the sensor was evaluated by measuring the inhibition effect of thioacetamide at a concentration of 0.1 μM. Between each measurement, the immobilized cells were washed with the Tris buffer solution and the growth media, respectively. As shown in Figure 5, the signal changes are fully reversible and the electrode can be repeatedly used for detection of toxicants.

To evaluate the accuracy of the biosensor, the colorimetric nitrite formation assay using cell suspensions of *N. europaea* was carried out. The inhibition percentages can be calculated as follows [13]:

(5) $$\text{inhibition}\%=(1-\frac{{\text{NO}}_{2\;\text{sample}}^{-}}{{\text{NO}}_{2\;\text{control}}^{-}})\times 100\%$$

**Figure 6.**
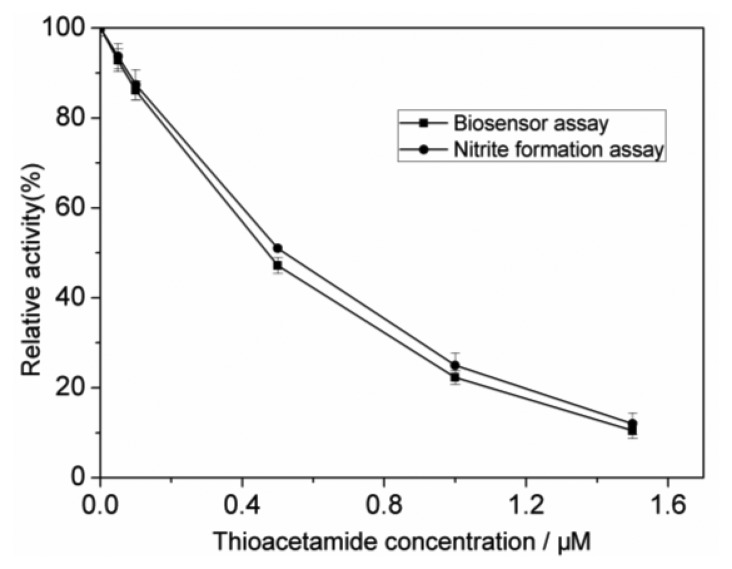
Inhibitory curves of thioacetamide at different concentrations obtained by the present biosensor (■) and the colorimetric nitrite formation assay (●) (relative activity% = 100%-inhibition%). Each error bar represents one standard deviation for three measurements.

As shown in Figure 6, the results obtained by the proposed biosensing flow system agree well with those obtained by the colorimetric method.

## 4. Conclusions

A microbial flow biosensor using a polymeric membrane ammonium-selective electrode as a transducer has been developed for detecting ammonia oxidation inhibitors. Unlike the conventional biosensing scheme, the proposed system allows the molecular recognition and transduction processes to be done individually, making the analytical procedures simple and convenient. The IC_50_ values have been measured as 0.17 μM and 0.46 μM for allylthiourea and thioacetamide, respectively. The present sensor offers advantages of simplicity, rapidity and high sensitivity for measuring toxicity in water.
